# The obesity-risk variant of FTO is inversely related with the So-Eum constitutional type: genome-wide association and replication analyses

**DOI:** 10.1186/s12906-015-0609-4

**Published:** 2015-04-15

**Authors:** Seongwon Cha, Hyunjoo Yu, Ah Yeon Park, Soo A Oh, Jong Yeol Kim

**Affiliations:** KM Health Technology Research Group, Medical Research Division, Korea Institute of Oriental Medicine, 1672 Yuseongdae-ro, Yuseong-gu, Daejeon, 305-811 Republic of Korea; Medical Engineering R&D Group, Medical Research Division, Korea Institute of Oriental Medicine, 1672 Yuseongdae-ro, Yuseong-gu, Daejeon, 305-811 Republic of Korea

**Keywords:** Genome-wide association study, FTO, Constitutional type

## Abstract

**Background:**

Body constitutional types described in the traditional Korean medicine system, Sasang constitutional medicine, are heritable, as has been revealed by twin and family studies. Thus, individuals with the same constitution type usually have similar pathophysiological and psychological traits. In several recent genome-wide association (GWA) analyses performed to identify constitution-associated variants, the association signals were not replicated due to small sample size and dissimilar, non-objective methods for classification of the constitutional types.

**Methods:**

We conducted GWA analysis and followed replication analysis in two large populations (5,490 subjects: 3,810 subjects at discovery stage and 1,680 subjects at replication stage) to identify the replicable constitution-associated variants, wherein subjects with the highest tertile of constitution probability values versus the reference with the lowest tertile of the values obtained from a recently developed constitution analysis tool were compared.

**Results:**

We found that the obesity-risk variant in intron 1 of the fat mass and obesity–associated (*FTO*) gene was replicably inversely associated with the So-Eum (SE) type, characterized by reduced appetite, slim body, and cautious personality (rs7193144 in combined samples: odds ratio = 0.729, *p* = 1.47 × 10^−7^), and substantial association signal remained after controlling for body mass index (BMI). In contrast, the association of the variant with the Tae-Eum type, characterized by high body mass, disappeared after controlling BMI.

**Conclusions:**

In summary, the obesity-risk variant in *FTO* intron 1 was inversely associated with the SE type, independent of BMI, which corresponded well with the characteristics of the SE type, such as the lowest body mass and lowest susceptibility to metabolic disorders among the constitutional types. Therefore, the obesity-risk variant of *FTO* associated with body mass increase might be involved in the determination of body constitution type.

**Electronic supplementary material:**

The online version of this article (doi:10.1186/s12906-015-0609-4) contains supplementary material, which is available to authorized users.

## Background

Sasang constitutional (SC) medicine—a Korean system of traditional medicine—categorizes human beings into four types (Tae-Eum (TE), So-Eum (SE), So-Yang (SY), and Tae-Yang (TY)) on the basis of responses to constitution-specified remedies [1]. Each SC type presents common characteristics of pathophysiological, psychological, and physical traits [2]. For instance, because the TE type has a tendency to gain weight, especially around the waist [3,4], people with this constitution show increased risks of cardiometabolic diseases such as metabolic syndrome, insulin resistance, hypertension, and diabetes mellitus [5-8]. In contrast, people with the SE type have slim body shape and reduced appetite and thus have the lowest susceptibility to cardiometabolic diseases among the SC types [4-11]. Compared to the SE type, SY type individuals are more active, easy-going, novelty seeking, and risk taking [12,13]. The cardiometabolic risk in the SY type is moderate, lying between that in the TE and SE types [7,14]. People with the TY type are considered to be creative, charismatic, slender waisted, and susceptible to musculoskeletal disease, but there has been very limited research on the TY type due to the very low proportion of Koreans with this constitution type (0.03–0.1%) [1].

These differences among SC types, especially for cardiometabolic risks between the TE and SE types, are attributed to the balanced status of physiological functions of the internal organs [1]. The TE type, with hyperactive function of fat deposition (also called liver function), is predisposed to risks of cardiometabolic diseases. In contrast, the SE type, with hypoactive digestive function (previously called spleen function), has low susceptibility to these diseases. Liver function is considered to be structurally compartmentalized into the liver and small intestine on the basis of the theory of SC medicine, and spleen function into the pancreas and stomach [15]. The four body compartmentalizations by physiological functions of representative visceral organs (lung, pancreas, liver, and kidney) according to the theory of SC medicine has been well explained by the modern concept of body patterning, e.g., anteroposterior axis patterning in the gastrointestinal tract modulated by *Hox* genes during the developmental stage [15,16]. Furthermore, body constitutional type is considered to be an inborn trait [1], a theory which is concurrent with the beliefs of Ayurveda [17], a traditional system of medicine native to India. Therefore, the differential characteristics among SC types might have an underlying genetic basis.

The heritability of SC types has been analyzed by family and twin studies [18,19], wherein the heritability values for TE, SE, and SY types from 101 pedigrees and 731 twin pairs were found to be 48–55%, 47%, and 41–43%, respectively. Several genome-wide association (GWA) analyses have been performed to elucidate genetic variations of SC types [20-23]. Two genetic loci, 8q11.22-23 and 11q22.1-3, were suggested as candidate loci linked to SC types in a genome-wide linkage analysis with a large family [22]. In addition, two GWA studies (GWASs) in unrelated populations comprising 60 and 1,222 individuals, respectively, listed constitution-associated variants [20,23], and the latter study attempted to find functional context of constitution-associated variants via text-mining–based and pathway-based network analyses [20,21].

However, previous genome-wide investigations have been limited by the lack of objectivity in classifying constitutional types and by the low reliability of the constitution-associated variants due to non-reproducibility of association signals among populations. The classification of SC type via self-assessment questionnaires and/or professionals is typically affected by subjectivity; therefore, it is necessary to develop a measurement-based objective tool for constitution typing. Recently, a diagnosis method for SC typing has been developed by integrating the holistic diagnostic processes of SC medicine practitioners and quantitative data on face, body shape, voice, and questionnaire information including personality traits [24]. The SC analysis tool (SCAT) is suggested to be a diagnostic model representing common rules among practitioners who have various points of view [24]. The current study aimed to discover the genetic variants reproducibly associated with constitutional types in two large populations, in which the subjects were classified into SC types by using the SCAT.

## Methods

### Subjects

The subjects for the GWAS were recruited as participants of community-based cohort studies from two regions in South Korea (Ansan and Ansung) from 2009 to 2012 for the Korean Genome and Epidemiology Study (KoGES) [25]. The criteria for inclusion of subjects in the study were availability of SCAT and genome-wide genotype data. The criteria for exclusion were as follows: a history of cancer; classification as a TY type, which is extremely uncommon in Korea (0.03–0.1%) [1]; low-quality genome-wide genotype data as previously described [25] including gender inconsistencies, cryptic relatedness, and problems with genotype call rate and sample contamination. In total, 5,716 individuals (2,681 men and 3,035 women) met the requirements and were selected for the study.

The participants were assigned probability values for each SC type. The SC types in this study were determined by the highest tertile of the probability values of SCAT for the corresponding constitutional type that had been classified by professionals of SC medicine [14,24]. That is, the subjects with the highest tertile of the SCAT probability values for the classification as TE type by professionals were designated as the TE type (n = 1,905), and the same process was used for designating the SE (n = 1,905) and SY types (n = 1,905). Furthermore, the subjects with the lowest tertile of the SCAT probability values for the TE, SE, and SY types determined by the professionals were classified and designated as non-TE (NTE; n = 1,905), non-SE (NSE; n = 1,905), and non-SY (NSY; n = 1,905), respectively. To increase the reliability for the SCAT-determined SC type, subjects with the middle tertile values were not used in the GWA analysis.

The 2,519 individuals (904 men and 1,615 women) for the replication analysis were recruited from 22 Oriental medical clinics for the Korea Constitution Multicenter Study (KCMS) from 2006 to 2012, after applying the above-mentioned inclusion and exclusion criteria except that for low-quality genotype data. The KCMS individuals were also classified on the basis of tertiles of the SCAT probability values for the SC type (TE, NTE, SE, NSE, SY, and NSY types; total, 840), and the individuals with middle tertiles were not used in the replication analysis. The KoGES and KCMS study participants were all of Korean ethnicity.

### Ethics statement

All subjects provided written informed consent to participate in the study, and the study was approved by the Institutional Review Board of the Korea Institute of Oriental Medicine.

### Genotyping

Genotyping of genomic DNA from the KoGES population was performed using Affymetrix Genome-Wide Human SNP (single nucleotide polymorphism) array 5.0 (Affymetrix, Santa Clara, CA); the details have been described in a previous report [25]. Among 500,568 SNPs in the Affymetrix SNP array, 311,944 SNPs were examined in the GWAS, since SNPs with high missing call rate (>5%), low minor allele frequency (< 0.05), and deviation from Hardy–Weinberg equilibrium (HWE; *p* < 0.0001) were excluded

The SE type–associated *FTO* variants from association analysis in KoGES population (GWAS and imputed SNP on chromosome 16), rs7193144, rs8050136, rs9939609, rs12149832, rs7185735, and so on (last two SNPs from imputation), were in perfect linkage disequilibrium (LD: *Dʹ* = 1.0, *r*^*2*^ = 1.0) with each other in the Asian HapMap population via Haploview program. Therefore, we used rs7193144 (as a representative of the GWAS variants) and rs7185735 (as a representative of the imputed variants: rs12149832 was deviated from HWE.) in the following analyses. The genotypes of rs7193144 in the KCMS population were determined by extracting the genotype from the Affymetrix SNP array for 557 subjects and by using an unlabeled oligonucleotide probe (UOP) on a polymorphic nucleotide for 1,962 subjects [26], whereas the genotypes of rs7185735 were determined by using UOP from 2,519 KCMS subjects. The detailed process of genotyping using a UOP for the variant has been described in a previous report [27]. The UOP (bold face for polymorphic site: 5′-GTTTAGT **C** GTTGAAATATGTTGTTTTGGTTGAAG-3′ for rs7193144 and 5′-CCCCGCTGAGGTAACTACCCC **C** ATGATAC-3′ for rs7185735) was made to span the variant by enabling it to form a perfectly matched duplex with 1 allele. The genomic template containing the variant was amplified by PCR with the following primer pair: forward primers for rs7193144 and rs7185735, 5′-CACATCTCTTTACTGTCTAGCTTG-3′ and 5′-TACTGGCATTTCTTCTTCACTG-3′, respectively; and reverse primers for rs7193144 and rs7185735, 5′-CACATCTCTTTACTGTCTAGCTTG-3′ and 5′-AAGAGACACCCAAGGTCTC-3′, respectively. An aliquot of the PCR amplicon including the SNP site was diluted in a solution containing 1 mM UOP, 5 mM SYTO-9 (Invitrogen, Carlsbad, CA), 12.5 mM EDTA, and 10 mM Tris (pH 8.0). The DNA in the UOP sample sequentially underwent denaturation (95°C for 5 s), annealing (60°C for 1 min), and melting with a gradual increase to 74°C at a rate of 1°C/s, while the fluorescence emission was read using the Light Cycler® 480 instrument (Roche, Indianapolis, IN). The genotype was determined from 3 melting patterns of the UOP (major homozygote, heterozygote, and minor homozygote).

### Statistical analysis

Not all subjects could be exclusively assigned to one specific SC type based on the SCAT data, especially in the case of the SE and SY types (Additional file 1: Figure S1). Therefore, the association between the SC type and the genetic variants was assessed using each binary SC variables (TE vs. NTE, SE vs. NSE, and SY vs. NSY), instead of using a single multinomial variable (TE, SE, and SY). Here, the NTE, NSE, and NSY were corresponding counter types of TE, SE, and SY, respectively. To compare characteristics of the study subjects between SC types and corresponding counter types, Mann–Whitney *U* test and chi-square test were used for continuous and categorical variables, respectively.

GWA analyses were performed for discovering variants associated with the TE type (vs. the NTE type), the SE type (vs. the NSE type), and the SY type (vs. the NSY type) using PLINK version 1.07 (http://pngu.mgh.harvard.edu/purcell/plink/) [28] by logistic regression analysis in an additive model, with adjustment for age and sex. A cut-off *p*-value was 5.0 × 10^−6^. Quantile–quantile plots for each SC type were constructed with the distribution of observed *p*-values against the theoretical distribution of expected *p*-values. The genomic control inflation factors (λ) for GWASs of each SC type were checked for potential *p*-value inflation. A regional association plot for a genomic region of 800 kb centered on the peak SNP was constructed using LocusZoom [29].

In the replication analysis, multiple logistic regression analysis was performed to determine the association of rs7193144 (a peak SNP from GWAS with the SE type) with the SC type, with adjustment for age and sex using R version 3.0.2 (http://www.r-project.org/). A cut-off *p*-value was 0.05. Chi-squared test was used to determine whether the GWAS SNP deviated from HWE in the KCMS population. LD (Lewontin’s *D’ = D/|D*_*max*_*|* and *r*^*2*^) was obtained using Haploview version 4.2 (Daly Lab at the Broad Institute, Cambridge, MA) [30].

The association results from the GWA and replication analyses were combined using Comprehensive Meta-Analysis program version 2.0 (Biostat, Englewood, NJ) in a random effect model using the DerSimonian and Laird method [31]. The SNP in combined analysis was considered significant, when *p*-values in GWA and replication analyses were all below the cut-offs (in GWAS: *p* < 5.0 × 10^−6^; in replication: *p* < 0.05).

### SNP imputation

We used imputed SNPs on chromosome 16, on which the *FTO* gene is located, provided by the Korea Center for Disease Control [25]. As shown in the previous study [25], SNP imputation was carried out using the IMPUTE software program [32] using JPT and CHB founders in HapMap as a reference panel (HapMap release 22, NCBI build 36, and dbSNP build 126). A total of 39,351 SNPs on chromosome 16 were obtained by combining imputed SNPs with the directly typed KoGES SNPs. After removing SNPs with minor allele frequency < 0.05, SNP missing rate > 0.05, and HWE *p* < 0.0001, we used the remaining 29,078 SNPs for association analysis of the SC types using the PLINK program.

## Results

We analyzed the common variants of the SC types in two Korean populations as follows: GWA analysis in a KoGES population comprising 5,716 individuals (discovery stage: stage 1) and replication analysis in a KCMS population comprising 2,519 individuals (replication stage: stage 2). The characteristics of all subjects, as well as subjects stratified by SC type, are presented in Table 1. The KoGES population included older individuals and a higher proportion of men than the KCMS population. As expected, the subjects with TE type had higher body mass index (BMI), waist circumference, blood pressure, and glucose and lipid levels compared to the other SC types. Physical and biochemical characteristics of the SC types (TE, SE, and SY) shown in Table 1 were different from those of the corresponding counter types (NTE, NSE, and NSY, respectively); the tendencies seen in the differences were as follows: TE type > NTE type, SE type < NSE type, and SY type < NSY type (Additional file 2: Table S1).

**Table 1:** **Characteristics of the subjects from two Korean populations stratified by Sasang constitutional type**

**Trait**	**KoGES**				**KCMS**			
	**All**	**TE**	**SE**	**SY**	**All**	**TE**	**SE**	**SY**
n	5,716	1,905	1,905	1,905	2,519	840	840	840
Age (y)	60.4 (8.5)	61.3 (8.6)	59.2 (8.3)	60.5 (8.6)	48.2 (15.9)	52.5 (16.0)	44.2 (15.1)	45.9 (15.2)
Woman (%)	53.1	48.7	60.4	52.7	64.1	53.9	68.9	72.9
Body mass index (kg/m^2^)	24.5 (3.4)	27.2 (3.5)	22.0 (2.2)	23.1 (2.3)	23.4 (3.3)	26.4 (2.8)	20.9 (2.4)	22.2 (2.5)
Waist circumference (cm)	86.5 (8.4)	93.3 (6.7)	80.0 (6.7)	82.9 (7.0)	84.0 (9.9)	92.5 (6.9)	76.9 (7.5)	79.6 (8.1)
HDL cholesterol (mg/dL)	45.9 (12.2)	43.9 (10.8)	48.2 (13.3)	46.5 (12.9)	48.0 (12.3)	43.3 (10.5)	51.8 (12.6)	51.0 (12.7)
LDL cholesterol (mg/dL)	119.8 (32.9)	119.6 (33.3)	119.9 (33.2)	119.5 (33.2)	108.5 (31.0)	113.9 (31.4)	102.6 (29.8)	104.7 (28.8)
Triglyceride (mg/dL)	142.6 (97.1)	155.8 (101.3)	131.3 (93.0)	138.8 (103.4)	124.9 (80.4)	153.5 (92.0)	99.0 (59.6)	109.9 (76.7)
Fasting blood glucose (mg/dL)	101.4 (25.3)	104.9 (27.4)	97.3 (19.6)	99.5 (23.6)	99.2 (28.2)	106.3 (34.9)	94.3 (21.8)	95.6 (24.8)
Systolic blood pressure (mmHg)	119.9 (16.8)	123.2 (16.0)	116.0 (16.6)	118.7 (17.3)	119.5 (15.9)	125.4 (15.2)	115.0 (14.7)	115.5 (15.7)
Diastolic blood pressure (mmHg)	77.6 (10.3)	79.4 (9.96)	75.4 (10.2)	76.7 (10.4)	76.9 (11.2)	80.6 (10.7)	74.1 (11.0)	74.3 (10.9)

Values are presented as mean (standard deviation) or as %.

Abbreviations: *KoGES* Korean Genome and Epidemiology Study, *KCMS* Korea Constitution Multicenter Study, *TE* Tae-Eum, *SE* So-Eum, *SY* So-Yang.

### Genome-wide association analysis for SC types (stage 1)

**Figure 1:**
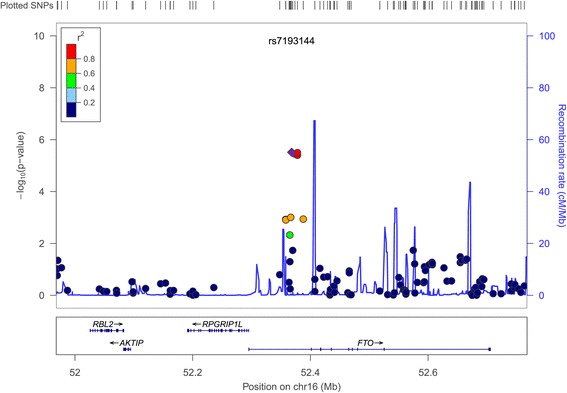
Regional association plot for *FTO* variants. The *FTO* variants associated with the So-Eum type (stage 1), in a genomic region of 800 kb centered on the peak variant rs7193144.

We performed a GWAS in the KoGES population to discover genetic variants determining the TE, SE, and SY types with reference to the corresponding NTE, NSE, and NSY types, respectively. The quantile–quantile plots for all three SC types demonstrated deviations between the distributions of expected and observed *p*-values only in the extreme tail probabilities (λ = 1.038 for TE type, λ = 1.031 for SE type, and λ = 1.012 for SY type) suggesting that population stratification had a negligible impact on the association results for each SC type (Additional file 3: Figure S2). No significant genome-wide association signals were found; however, SNPs on chromosome 16 showed strong association with the SE type (*p* < 5.0 × 10^−6^) (Additional file 4: Figure S3). Interestingly, the variants associated with the SE type were located in intron 1 of *FTO*, as shown in the regional association plot (Figure 1 and Additional file 5: Table S2). The SE type was associated with the decreased number of minor alleles of the *FTO* variant (rs7193144: odds ratio (OR) = 0.714, 95% confidence interval (CI) = [0.620–0.823]) (Table 2). The variant showed moderate association with the TE type, with opposite effects compared to the SE type (rs7193144: OR = 1.224, 95% CI = [1.067–1.405]), whereas there were no associations with the SY type (Table 2).

**Table 2:** **Association of** ***FTO*** **rs7193144 (T > C) with determination of constitutional type**

**Population**	**SE versus NSE**				**TE versus NTE**				**SY versus NSY**			
	**MAF (n)**				**MAF (n)**				**MAF (n)**			
	**SE**	**NSE**	**OR (95% CI)**	***P***	**TE**	**NTE**	**OR (95% CI)**	***P***	**SY**	**NSY**	**OR (95% CI)**	***P***
Stage 1	0.106 (1903)	0.140 (1903)	0.714 (0.620, 0.823)	3.07E-06	0.135 (1903)	0.114 (1903)	1.224 (1.067, 1.405)	0.00395	0.122 (1902)	0.131 (1903)	0.918 (0.801, 1.053)	0.222
Stage 2	0.122 (833)	0.146 (835)	0.764 (0.616, 0.945)	0.0135	0.146 (837)	0.119 (831)	1.422 (1.144, 1.772)	0.00161	0.119 (831)	0.137 (835)	0.820 (0.664, 1.013)	0.0667
Combined	0.111 (2736)	0.142 (2738)	0.729 (0.648, 0.820)	1.47E-07	0.138 (2740)	0.116 (2734)	1.287 (1.120, 1.478)	3.87E-05	0.121 (2733)	0.133 (2738)	0.888 (0.792, 0.996)	0.0585
Heterogeneity	Q = 0.293, *p* = 0.588, I^2^ = 0				Q = 1.194, *p* = 0.275, I^2^ = 16.22				Q = 0.834, *p* = 0.361, I^2^ = 0			

Abbreviations: *FTO* fat mass and obesity–associated, *SE* So-Eum, *NSE* non-So-Eum, *TE* Tae-Eum, *NTE* non-Tae-Eum, *SY* So-Yang, *NSY* non-So-Yang, *MAF* minor allele frequency, *OR* odds ratio, 95% CI, 95% confidence interval.

Logistic regression analysis with adjustment for age and sex in an additive genetic model: (stage 1) genome-wide association analysis in the KoGES population; (stage 2) replication analysis in the KCMS population.

### Replication (stage 2) and combined analysis (stage 1 and 2)

To confirm the association of the *FTO* variant rs7193144 with the SE type, we performed replication analysis (stage 2) in the KCMS population with reference to the NSE type. The association of the SE type with rs7193144 was confirmed (OR = 0.764, 95% CI = [0.616–0.945]), and the variant was also found to be associated with the TE type (OR = 1.422, 95% CI = [1.144–1.772]) (Table 2). When combining the effects both in stage 1 and 2, rs7193144 was associated both with the SE (significantly) and TE types (moderately) with completely opposite effects (SE type: combined OR = 0.729, 95% CI = [0.648–0.820]; TE type: combined OR = 1.287, 95% CI = [1.120–1.478]) (Table 2).

The effects of the *FTO* variant rs7193144 on the SC type were re-analyzed after adjusting for BMI, as the characteristics of each constitutional type (TE, SE, and SY) were different from those of the corresponding counter types (NTE, NSE, and NSY). Interestingly, the association of rs7193144 with the TE type disappeared after controlling for BMI in the stage 1, 2, and 1 + 2 populations (Table 3). Therefore, the association of the TE type with the *FTO* variant might be mediated by BMI, as it is usually higher in the TE than in the other types (Table 1). However, the association of the *FTO* variant with the SE type remained after controlling for BMI in the stage 1, 2, and 1 + 2 populations (combined analysis: OR = 0.693, 95% CI = [0.572–0.839]), although the effect diminished slightly (Table 3). Thus, the *FTO* variant was found to be associated with the SE type, independent of BMI.

**Table 3:** **Associations of** ***FTO*** **rs7193144 (T > C) with determination of So-Eum or Tae-Eum type, after adjusting age, sex, and BMI**

**Population**	**SE versus NSE**				**TE versus NTE**			
	**MAF (n)**				**MAF (n)**			
	**SE**	**NSE**	**OR (95% CI)**	***P***	**TE**	**NTE**	**OR (95% CI)**	***P***
Stage 1	0.106 (1903)	0.140 (1903)	0.706 (0.514, 0.968)	0.00210	0.135 (1903)	0.114 (1903)	1.097 (0.856, 1.407)	0.466
Stage 2	0.122 (833)	0.146 (835)	0.706 (0.514, 0.967)	0.0308	0.146 (837)	0.119 (831)	1.248 (0.885, 1.762)	0.207
Combined	0.111 (2736)	0.142 (2738)	0.693 (0.572, 0.839)	1.73E-04	0.138 (2740)	0.116 (2734)	1.146 (0.937, 1.402)	0.183

Abbreviations: *FTO* fat mass and obesity–associated, *BMI* body mass index,* SE* So-Eum, *NSE* non-So-Eum, *TE* Tae-Eum, *NTE* non-Tae-Eum, *MAF* minor allele frequency, *OR* odds ratio, 95% CI, 95% confidence interval.

Logistic regression analysis with adjustment for age, sex, and BMI in an additive genetic model: (stage 1) genome-wide association analysis in the KoGES population; (stage 2) replication analysis in the KCMS population.

### SNP imputation on chromosome 16

We re-analyzed the GWAS data after SNP imputation on chromosome 16, which contains the *FTO* gene. As shown in Additional file 6: Table S3 and Additional file 7: Figure S4, the association signals of rs7185735 for the SE type (OR = 0.729, 95% CI = [0.647–0.821]) showed similar association trends as rs7193144 (OR = 0.729, 95% CI = [0.648–0.820]) in combined population. However, after adjusting for BMI, rs7185735 was not associated with the SE type in the KCMS population (*p* > 0.05) (Additional file 6: Table S3). For the TE and SY types, the association trends of rs7185735 were exactly the same as those of rs7193144 (Additional file 6: Table S3).

## Discussion

SC medicine is already tailored to an individual patient using constitution-specified herbal remedies on the basis of SC type [1], whereas occidental medicine can provide drug therapy tailored to the patient only after evaluating pharmacogenomic variability for an individual drug [33]. We found that the *FTO* variant, rs7193144, was inversely associated with the SE type versus the NSE type. Therefore, elucidation of genetic variations determining SC types, such as an *FTO* variant with the SE type in this study, can provide individualized herbal remedies akin to modern personalized medicine.

SNP rs7193144 was reliably associated with the SE type in two populations, independently of the BMI. The BMI was adjusted in the association between the *FTO* variant and SC types, since the rs7193144 is strongly linked with rs9939609 and rs8050136 variants in the Asian HapMap population which variants are well known to contribute to obesity and diabetes through affecting BMI [34-36]. After imputation of SNPs on chromosome 16, we found strong association with SE type for SNP rs7185735, which is in strong LD with rs7193144 (*Dʹ* = 1.0 and *r*^*2*^ = 1.0 in Asian HapMap population). The trends of association signals of rs7185735 were similar to those of rs7193144 for the three SC types. However, after adjusting for BMI, the association of rs7185735 with the SE type was weak. The deviating signal for rs7185735 may be due to the inference of the genotype by imputation instead of by direct genotyping. Therefore, we hypothesize that rs7193144 might be a replicable and independent variant associated with the SE type.

The data used in this study were more reliable than those of previously reported GWASs for SC types in two aspects [20,23]: (1) objectivity in classifying the SC types by using SCAT, an integrated diagnostic model consisting of quantitative assessments of face, body shape, and voice and questionnaire information including personality traits, and (2) reliability in the association results by confirming the GWAS results in another independent population and by excluding the subjects with the middle tertile of SCAT probability values to rule out ambiguous classification into the SC types. We could not find replicable variants for the SC types with the two previous GWA analyses, although a variant was located in the same genomic locus containing the *FTO* variants [23]. The *FTO* variants located on locus 16q12.2 associated with determination of the SE type are adjacent to a variant (rs12599068) of the iroquois homeobox 3 gene, which has been suggested to have an effect on the constitutional type [23]. However, it cannot be conclusively stated that the previously reported constitution-associated variant was replicated in this study, since the *FTO* variant and the iroquois homeobox 3 variant are too distant (approximately 560 kb apart: *Dʹ* = 0.07 in Asian HapMap population) to be linked to each other.

The reported characteristics, namely, body shape, psychological traits, appetite, and disease risk, of the SE type in the current study were inversely related to the traits associated with the obesity-risk alleles of *FTO*. Since the obesity-risk alleles of *FTO* have been found to affect weight gain [34,35], dietary intake [37,38], and metabolic disorders [36,39,40], the lower carriage of the risk allele in the subjects with the SE type explains why this SC type has a propensity to have a relatively lower prevalence of abdominal obesity [4], lower appetite [10,11], and lower risks of cardiometabolic diseases than other SC types [5-8]. As regards psychological traits, the *FTO* alleles are highly expressed in the hypothalamus of the central nervous system and are known to be related with behavioral phenotypes such as increased food impulsivity and better emotional control in childhood [41], which appears to be conflicting with the personality of the SE type. The SE type is associated with low novelty-seeking and high harm-avoidance scores in the temperament dimension; passive, static, and meticulous tendencies in the Sasang personality questionnaire; and high neuroticism and low extraversion scores [12,13,42]. Therefore, we suggest that the neuronal action of the *FTO* allele on behavior and metabolism for inducing weight gain may affect the psychological characteristics and bodily structure of the SE type, such as cautious personality and slim body. Moreover, the effect of the *FTO* allele is manifested from infancy till adulthood [43,44]; thus, it supports the hypothesis that the SC type is an inborn trait.

The limitation of this study is that the constitution-associated variants were identified at a very low rate despite the >40% heritability of the SC type [2,18,19], i.e., only one locus for the SE type and no locus for TE and SY types under *p* < 5.0 × 10^−6^. Therefore, it is impossible to classify an individual’s SC type only with genetic variations. The main reason for the lack of associations might be the small sample size, which implies that the SC types are too polygenic to identify the constitution-associated SNPs in the studied populations. Therefore, it will be necessary to use an even larger population or focus on more specific traits comprising the SC types in order to identify more constitution-associated variants. In addition, additional studies may be necessary to account for the missing heritability of the SC type with many mild-effect variants in a genome-wide SNP microarray using, for instance, a tool for genome-wide complex trait analysis [45,46]. It is also interesting to note that demethylation of m^6^A by FTO has been discovered, underscoring the importance of this gene in RNA biology [47]. Further studies will be needed to investigate effects of epigenetic regulation in the context of constitutional types, especially in the SE type.

## Conclusions

In conclusion, we elucidated that the obesity-risk variant of *FTO*, rs7193144, was inversely associated with the SE type constitution, independent of BMI, on the basis of SCAT probability values from two-stage analyses of GWAS and replication analysis in Koreans. The lower predispositions to weight gain and metabolic disorders corresponded well with the *FTO* risk allele non-carriers compared to the carriers. Therefore, the obesity-risk variant of *FTO* might be involved not only in fat mass accumulation but also in constitution typing.

## Additional files

Additional file 1: Figure S1. SC typing patterns of study participants. Additional file 2: Table S1. Differences in characteristics between SC types and corresponding counter types (TE vs. NTE, SE vs. NSE, and SY vs. NSY) Values are presented as mean (standard deviation) or as %. Abbreviations: KoGES, Korean Genome and Epidemiology Study; KCMS, Korea Constitution Multicenter Study; TE, Tae-Eum; SE, So-Eum; SY, So-Yang; NTE, non-Tae-Eum; NSE, non-So-Eum; NSY, non-So-Yang. *Continuous traits: Mann–Whitney *U* test; categorical traits: chi-square test. Additional file 3: Figure S2. Quantile–quantile plots for constitutional types. The quantile–quantile plots of the observed *p*-values of variants from genome-wide association analysis for each constitutional type versus the expected *p*-values. (A) Tae-Eum type (λ = 1.038), (B) So-Eum type (λ = 1.031), and (C) So-Yang type (λ = 1.012). Additional file 4: Figure S3. Manhattan plots for constitutional types. The Manhattan plots of the *p*-values (−log_10_(*P*)) of variants from genome-wide association analysis for each constitutional type in the whole chromosomal region (chromosomes 1–22): the blue line indicates a *p*-value of 5.0 × 10^−6^. (A) Tae-Eum type, (B) So-Eum type, and (C) So-Yang type. Blue arrowhead with *p* < 5.0 × 10^−6^. Additional file 5: Table S2. Genome-wide association analysis to determine the SE type in the Korean Genome and Epidemiology Study population (*p* < 5.0 × 10^−6^). Additional file 6: Table S3. Association of FTO rs7185735 (A > G) with determination of constitutional type. Abbreviations: FTO, fat mass and obesity–associated; SE, So-Eum; NSE, non-So-Eum; TE, Tae-Eum; NTE, non-Tae-Eum; SY, So-Yang; NSY, non-So-Yang; MAF, minor allele frequency; OR, odds ratio; 95% CI, 95% confidence interval. Logistic regression analysis with adjustment for age and sex in an additive genetic model: (stage 1) genome-wide association analysis in the KoGES population; (stage 2) replication analysis in the KCMS population. Additional file 7: Figure S4. Regional association plot for an imputed *FTO* variant. The *FTO* variant associated with the So-Eum type, in a genomic region of 800 kb centered on the peak variant rs7185735.

## Abbreviations

BMI: Body mass index
95% CI: 95% Confidence interval
FTO: Fat mass and obesity-associated gene
GWAS: Genome-wide association study
HWE: Hardy-Weinberg equilibrium
KCMS: Korea constitution multicenter study
KoGES: Korean genome and epidemiology study
LD: Linkage disequilibrium
NSE: Non-So-Eum
NSY: Non-So-Yang
NTE: Non-Tae-Eum
OR: Odds ratio
SCAT: Sasang constitutional analysis tool
SNP: Single nucleotide polymorphism
TY: Tae-Yang
UOP: Unlabeled oligonucleotide probe
