# Supplementary figures and images for: The obesity-risk variant of FTO is inversely related with the So-Eum constitutional type: genome-wide association and replication analyses
Source: BMC Complement Altern Med. 2015 Apr 15;15:120. doi: 10.1186/s12906-015-0609-4 (PMC4432511; doi:10.1186/s12906-015-0609-4)

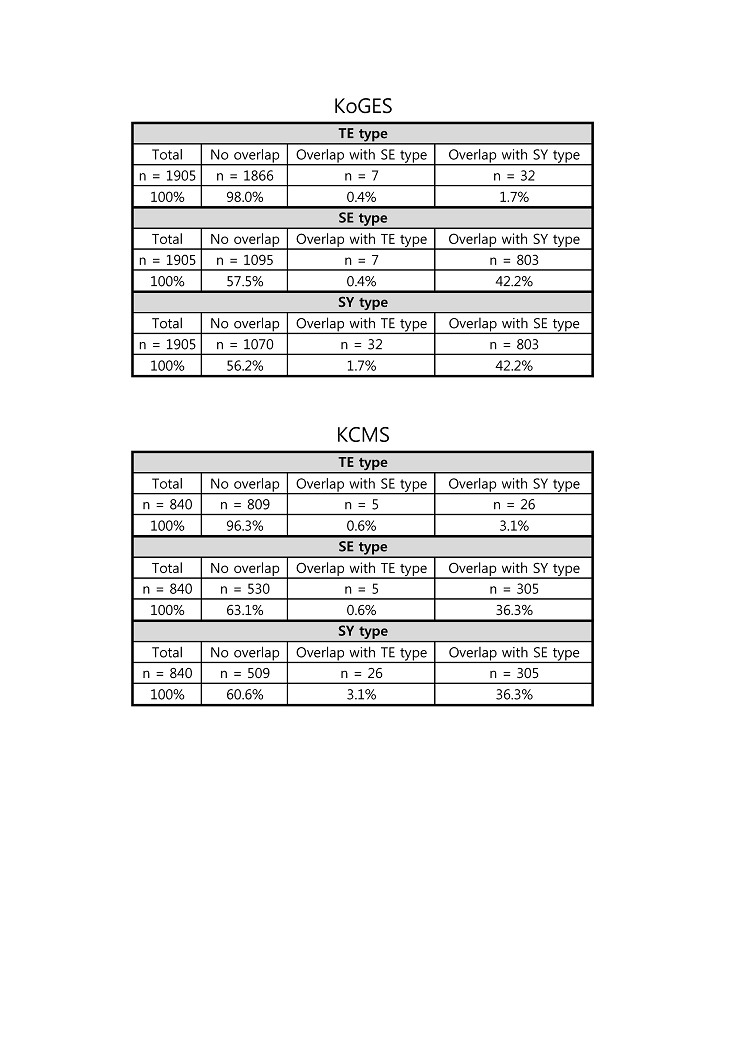

Supplement: Additional file 1: Figure S1. — SC typing patterns of study participants. [file 12906_2015_609_MOESM1_ESM.tiff]

## Slide 1
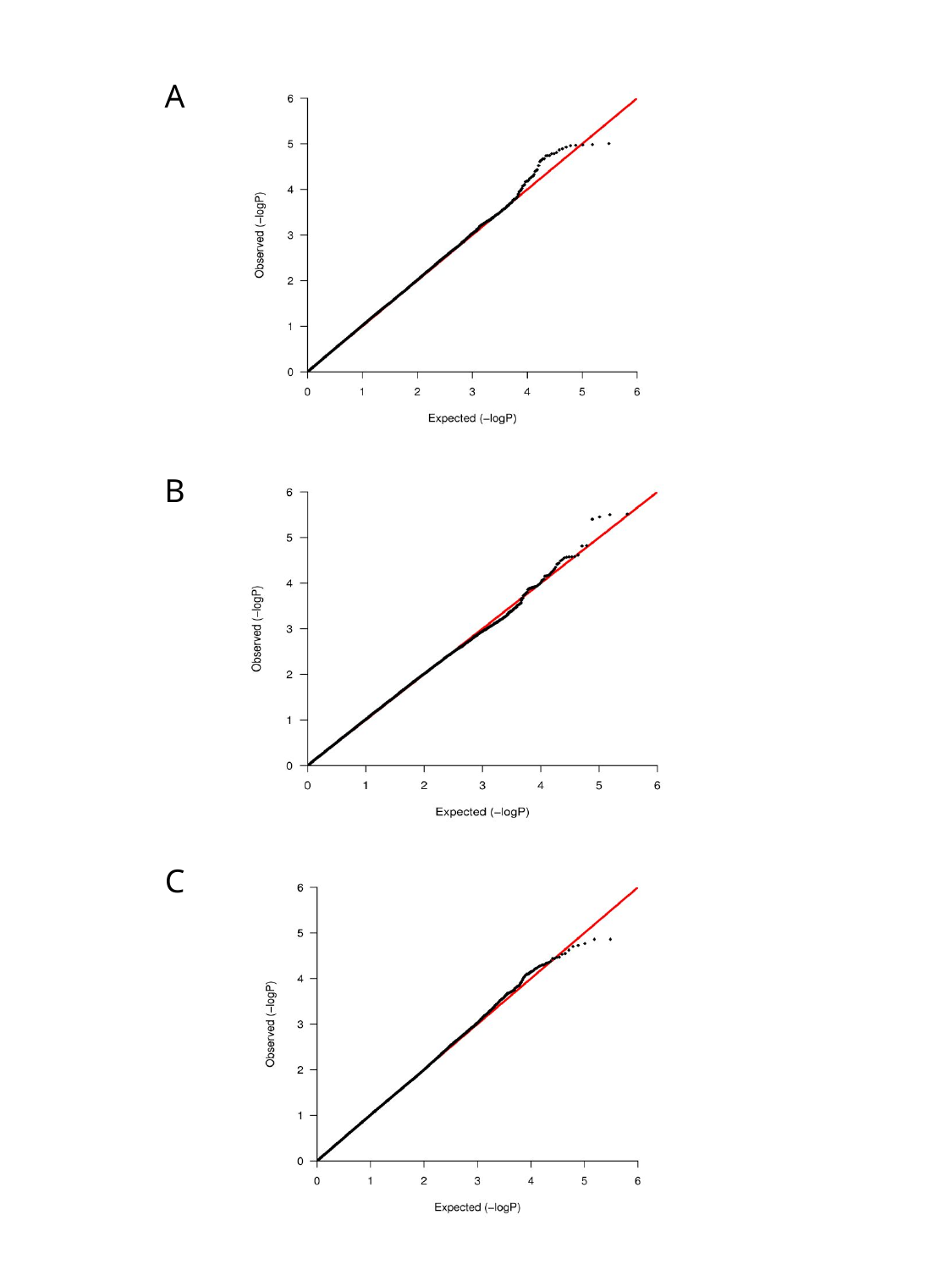

A
B
C

Supplement: Additional file 3: Figure S2. — Quantile–quantile plots for constitutional types. The quantile–quantile plots of the observed p-values of variants from genome-wide association analysis for each constitutional type versus the expected p-values. (A) Tae-Eum type (λ = 1.038), (B) So-Eum type (λ = 1.031), and (C) So-Yang type (λ = 1.012). [file 12906_2015_609_MOESM3_ESM.pptx]

## Slide 1
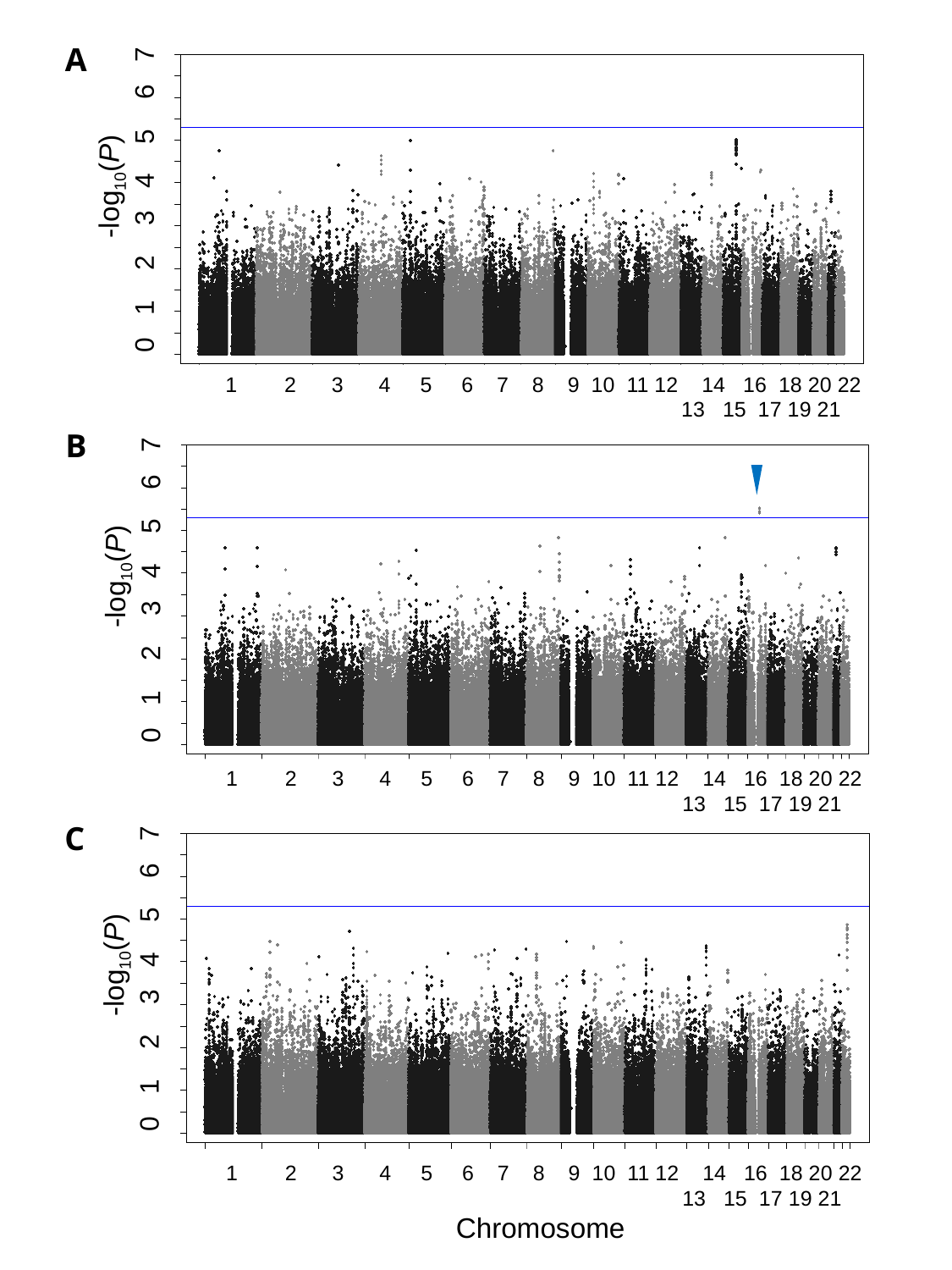

-log10(P)
0 1 2 3 4 5 6 7
A
 1 2 3 4 5 6 7 8 9 10 11 12 14 16 18 20 22
 13 15 17 19 21
-log10(P)
0 1 2 3 4 5 6 7
B
 1 2 3 4 5 6 7 8 9 10 11 12 14 16 18 20 22
 13 15 17 19 21
-log10(P)
0 1 2 3 4 5 6 7
C
 1 2 3 4 5 6 7 8 9 10 11 12 14 16 18 20 22
 13 15 17 19 21
Chromosome

Supplement: Additional file 4: Figure S3. — Manhattan plots for constitutional types. The Manhattan plots of the p-values (−log10(P)) of variants from genome-wide association analysis for each constitutional type in the whole chromosomal region (chromosomes 1–22): the blue line indicates a p-value of 5.0 × 10^−6^. (A) Tae-Eum type, (B) So-Eum type, and (C) So-Yang type. Blue arrowhead with p < 5.0 × 10^−6^. [file 12906_2015_609_MOESM4_ESM.pptx]

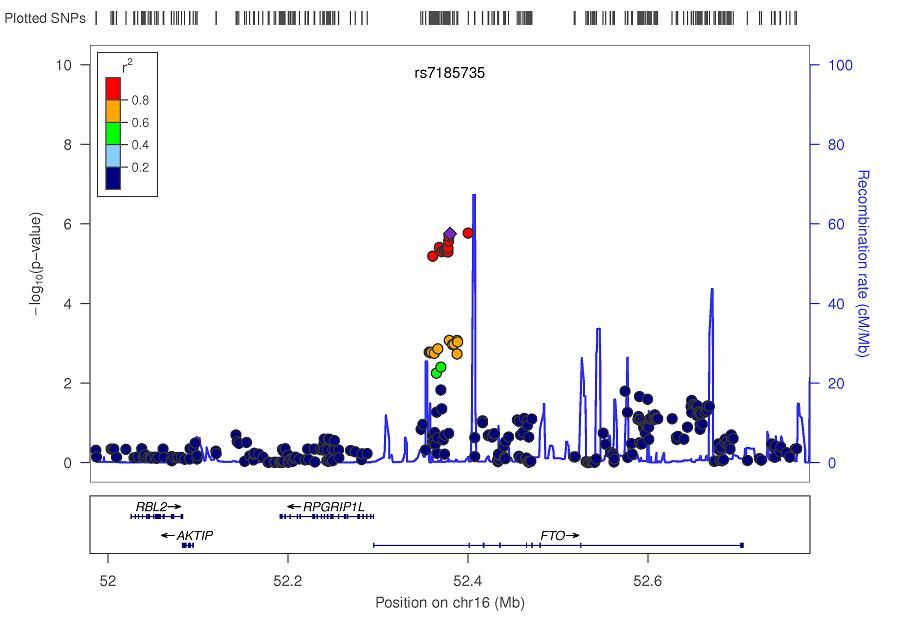

Supplement: Additional file 7: Figure S4. — Regional association plot for an imputed FTO variant. The FTO variant associated with the So-Eum type, in a genomic region of 800 kb centered on the peak variant rs7185735. [file 12906_2015_609_MOESM7_ESM.tiff]
